# Correlation between increased total serum IgE levels and clinical features in alopecia areata patients

**DOI:** 10.3389/fimmu.2025.1581976

**Published:** 2025-06-02

**Authors:** Yijie Sun, Shuang Xu, Yujing Zhang, Yuxin Wang, Jianzhong Zhang, Xiangqian Li, Cheng Zhou

**Affiliations:** ^1^ Peking University People’s Hospital, Department of Dermatology, Beijing, China; ^2^ Peking University People’s Hospital, Department of Clinical Laboratory, Beijing, China

**Keywords:** alopecia areata, IgE, Th2 pathway, cytokines, alopecia universalis, alopecia totalis

## Abstract

**Backgrounds:**

Alopecia areata (AA) is primarily associated with a Type 1 (Th1) inflammatory response, but emerging evidence also suggests a potential contribution of Type 2 (Th2) immunity. However, the relationship between IgE levels and pathogenesis of AA, as well as its clinical features, remains unclear, with limited and conflicting evidence in current research. This study aims to compare the total serum IgE levels between AA patients without atopic diseases and healthy controls, and to examine the correlation of IgE levels with gender, age, disease severity, disease duration, and affection of eyebrows, eyelashes, and nails.

**Methods:**

AA patients without other hair loss diseases and conditions, or systemic treatment known to affect serum IgE were included. Medical records of 436 patients and 181 normal controls were retrospectively analyzed between May 2018 and November 2024. Among AA patients, clinical features and total serum IgE levels were analyzed.

**Results:**

The elevated total serum IgE rate was observed in 31.0% of AA patients, which was significantly higher than that in control groups (P < 0.001). Among AA patients, total serum IgE levels differed significantly by gender (P = 0.002), age (P < 0.001), and disease severity (P = 0.040). Patients with alopecia universalis or totalis (AU/AT) had higher total serum IgE levels compared to those with localized AA (P = 0.049). No significant correlation was found between total serum IgE levels and disease duration.

**Conclusions:**

Our research reveal that total serum IgE levels are elevated in AA patients compared to control group. Male patients, children, and individuals with moderate-severe AA patients showed significantly higher IgE levels. These results suggest the involvement of IgE and Th2 cytokines in AA pathogenesis.

## 1 Introduction

Alopecia areata (AA) is a non-scarring inflammatory hair loss disease with a prevalence of approximately 2%, showing no significant gender differences. AA typically presents with well-defined patches of hair loss on the scalp and may progress to involve the entire scalp (alopecia totalis) or even the entire body (alopecia universalis) in severe cases, which has profound psychological impact on the patients.

Although the exact etiology of AA remains unclear, the collapse of immune privileged of hair follicle is considered the primary pathogenesis of AA (1). CD8+ NKG2D+ T cells and Th1 inflammatory responses are considered as the primary pathogenesis, with interferon-gamma (IFN-γ) being a key inflammatory mediator (2). However, some studies have shown that both Th1 (such as IFN-γ) and Th2 (such as IL-4 and IL-10) cytokines are elevated in AA patients, suggesting that Th2 cytokines might play a role in the inflammatory process of AA (3). Immunoglobulin E (IgE) is one of the fewest secreted isotypes of immunoglobulins but can be significantly elevated in allergic diseases, such as atopic dermatitis. Some studies have observed an increased incidence of AA in patients with a history of allergic diseases (4). Additionally, compared to patients with late-onset or mild AA, those with early-onset or severe AA might show higher total IgE levels (5). IgE secretion is mediated by Th2 cytokines, activated ILC2 cells and Th2 cells produce Th2 cytokines like IL-4 and IL-13, which promote antibody class-switching from IgM to IgE (6). Moreover, dupilumab, an IL-4 receptor alpha (IL-4Rα) inhibitor, has been clinically evaluated for the treatment of both adult and pediatric AA patients (7–9), and shown greater efficacy in AA patients whose IgE levels exceed 200 IU/mL compared to those with lower IgE levels, which also support the important role of Th2 cytokines in the AA pathogenesis (10, 11).

Despite these findings, data on the relationship between serum IgE levels and the risk and clinical features of AA is controversial, largely due to small sample sizes and variations in study population and design. Hence, this study aims to compare the total serum IgE levels between AA patients without atopic diseases and healthy individuals, and to evaluate the associations between total serum IgE levels and clinical features, including age, gender, AA subtypes, disease severity, disease duration, and involvement of other body hairs and nails.

## 2 Methods

Ethical approval for this retrospective study was obtained. Case of AA patients and age- and gender-matched health control between May 2018 and November 2024 were collected from the hospital database.

Patients meeting the following criteria were included:

1. Diagnosed with AA based on typical clinical manifestations and trichoscopic findings, with no other concurrent hair loss diseases;
2. At the time of IgE measurement, patients had not received systemic treatment for AA within the past six months, including corticosteroids, immunosuppressants, JAK inhibitors, dupilumab, or any other treatments that could affect total serum IgE levels;
3. We conducted comprehensive reviews of medical history and diagnostic records. Patients with known diseases affecting total serum IgE levels such as allergic diseases or parasitic infections were excluded.

Patients with the following criteria were excluded:

1. Incomplete clinical information, such as missing data on AA subtype, disease duration, or disease severity.

Controls meeting the following criteria were included:

1. No history of AA or diseases known to affect total serum IgE levels, such as allergic diseases or parasitic infections;
2. Matched with gender and age to the AA patients.

Clinical data from AA patients were documented, including demographic data (gender and age), clinical features (disease severity, disease duration, AA subtype, and involvement of eyebrows, eyelashes, and nails), and total serum IgE levels. Disease duration was defined as the time from the first onset of AA to the time of serum IgE testing at the current visit.

The severity of AA was graded based on the Severity of Alopecia Tool (SALT) score: mild (SALT score < 25), moderate (25 ≤ SALT score < 50), and severe (SALT score ≥ 50). The mild cases were stratified into a separate group, while moderate and severe cases were combined into a single group.

Total serum IgE levels were measured using electrochemiluminescence immunoassay for the quantitative determination of IgE concentration in human serum (Roche cobas e801, Roche Diagnostics, Germany). In accordance with the reagent instruction, an IgE level of ≥100 IU/mL was considered elevated.

Continuous variables were described as mean ± standard deviation, while categorical variables were described as frequencies and percentages. T-tests were used for inter-group comparisons of continuous variables with a normal distribution. Mann-Whitney U test or Kruskal-Wallis test was used for non-normally distributed continuous variables. Chi-square test was used for comparisons of categorical variables. Binary logistic regression analysis was conducted to evaluate the independent effects of clinical covariates. Statistical analyses were performed using SPSS version 26.0, with a significance level of *P* < 0.05.

## 3 Results

### 3.1 Demographic characteristics of AA patients and controls

This study included 436 AA patients and 181 age- and gender-matched healthy controls. The mean age of the AA patients was 33.07 ± 14.36 years, with 167 (38%) male patients and 269 (62%) female patients. The mean age of the control group was 31.27 ± 11.81 years, with 67 (37%) male patients and 114 (63%) female patients. No significant differences were found between the two groups on age (*P* = 0.066) and gender (*P* = 0.764).

The mean disease duration of AA patients was 37.50 ± 59.11 months. Among the AA patients, 187 (43%) had mild disease, and 249 (57%) had moderate-to-severe disease. The distribution of AA subtypes was as follows: 341 (78%) with patchy AA, 48 (11%) with AU/AT, 15 (3%) with acute diffuse and total alopecia (ADTA), and 32 (7%) with ophiasis subtype. 102 (23%) patients had involvement of other body areas. The clinical characteristics of the AA patients were summarized in **Table 1**.

**Table 1:** Summary of clinical data of the AA patients.

Variables	AA group
Age, years ± SD	33.07 ± 14.36
Gender, n pts (%)	
Male	167 (38)
Female	269 (62)
Mean AA duration, months ± SD	37.50 ± 59.11
Severity, n pts (%)	
Mild	187 (43)
Moderate to severe	249 (57)
AA subtypes, n pts (%)	
LAA	341 (78)
AU/AT	48 (11)
ADTA	15 (3)
Ophiasis	32 (7)
Eyebrow/eyelash and nail affection, n pts (%)	
Positive	102 (23)
Negative	334 (77)

AA, alopecia areata; AU/AT, alopecia universalis or alopecia totalis; ADTA, acute diffuse and total alopecia; LAA, localized alopecia areata.

### 3.2 Comparison of serum IgE levels between AA patients and controls

The mean total serum IgE level in AA patients was 108.87 ± 163.26 IU/mL (range: 0.43-854.9 IU/mL), while in the control group, it was 61.19 ± 76.89 IU/mL. A significant difference in serum IgE levels was observed between the two groups (U = 47456, *P* < 0.001).

Among the AA patients, 135 (31%) patients had serum IgE levels ≥100 IU/mL, while 301 (69%) had levels <100 IU/mL. In AA patients with IgE levels ≥100 IU/mL, the gender ratio of was 1:1.5, with a mean age of 31.10 years, while in AA patients with IgE <100 IU/mL, the gender ratio of was 1:1.9, with a mean age of 33.95 years. Significant differences were observed in gender (*P* = 0.009) and age (*P* = 0.021) between the two groups. However, there were no significant differences in disease severity (*P* = 0.062), disease duration (*P* = 0.877), or involvement in other areas (*P* = 0.280).

### 3.3 Correlation between clinical characteristics of AA and total serum IgE levels

Total serum IgE levels were significantly higher in male AA patients than female (133.96 ± 198.44 IU/mL vs. 93.30 ± 135.03 IU/mL, *P* = 0.008), the ratio of elevated IgE is 38.3% in male patients vs. 26.4% in female patients (*P* = 0.009). The mean total serum IgE level in pediatric AA patients was 190.24 ± 279.32 IU/mL, which was significantly higher than adult AA patients (94.36 ± 127.60 IU/mL, *P* < 0.001), the ratio of elevated IgE is 43.9% in pediatric patients vs. 28.6% in adult patients (*P* = 0.013). Patients with moderate-to-severe AA had a higher mean total serum IgE level (129.00 ± 195.90 IU/mL) than those with mild AA (82.07 ± 99.50 IU/mL, *P* = 0.017), no significant difference was observed in the ratio of elevated IgE between moderate-to-severe patients and mild patients (34.5% vs. 26.2%, *P* = 0.062). Additionally, no significant correlations were found between total serum IgE levels and disease duration (*P* = 0.171) or other areas of involvement (*P* = 0.211).

Significant differences were observed in serum IgE levels among different AA subtypes. The mean serum IgE level was as follows:

AU/AT: 180.79 ± 223.87 IU/mL

Patchy alopecia areata: 98.85 ± 132.15 IU/mL

Ophiasis: 114.80 ± 306.42 IU/mL

ADTA: 94.05 ± 85.57 IU/mL

Among these, only patients with AU/AT had significantly higher serum IgE levels than those with patchy AA (*P* = 0.049), while there were no significant differences between other subtypes. These results were summarized in **Table 2**.

**Table 2:** Association between IgE levels and clinical parameters of AA patients.

Clinical parameters	Mean ± SD	Test of significance	*P* value
Gender			
Male (n=167)	133.96 ± 198.44	U test	0.002*
Female(n=269)	93.30 ± 135.03		
Age			
≤18 years(n=66)	190.24 ± 279.32	U test	<0.001*
>18 years(n=370)	94.36 ± 127.60		
Duration			
≤2 year(n=285)	99.76 ± 121.19	U test	0.171
>2 year(n=151)	126.08 ± 221.43		
Severity			
Mild(n=187)	82.07 ± 99.50	U test	0.040*
Moderate to severe(n=249)	129.00 ± 195.90		
Type of AA			
LAA	98.85 ± 132.15	K test	0.033*
AU/AT	180.79 ± 223.87		
ADTA	94.05 ± 85.57		
Ophiasis	114.80 ± 306.42		
Eyebrow/eyelash and nail affection			
Positive(n=102)	140.94 ± 234.99	U test	0.211
Negative(n=334)	99.08 ± 132.88		

AA, alopecia areata; AU/AT, alopecia universalis or alopecia totalis; ADTA, acute diffuse and total alopecia. IgE, Immunoglobulin E; LAA, localized alopecia areata; U test, Mann-Whitney test; *: Significant.

In AU/AT patients, total serum IgE levels were significantly higher in pediatric AA patients than adult patients (U = 125, *P* = 0.06), no significant correlation was found between total serum IgE levels and gender (U = 199, *P* = 0.079) and disease duration (U = 296, *P* = 0.869).

In LAA patients, total serum IgE levels were significantly higher in male AA patients than female patients (U = 11809, *P* = 0.014), no significant correlation was found between total serum IgE levels and age (U = 5600.5, *P* = 0.057) and disease duration (U = 10795, *P* = 0.152).

A multivariate regression suggested that among gender, age, severity, duration of AA, AA subtype, and involvement of eyebrows, eyelashes, and nails, only gender was a significant factor of IgE (*P* = 0.012).

### 3.4 Clinical characteristics of AA patients with serum IgE levels ≥200 IU/mL

There were 73 AA patients with total serum IgE levels ≥200 IU/mL and 363 patients with total serum IgE levels <200 IU/mL. Among those with IgE levels ≥200 IU/mL, the male-to-female ratio was 1:0.97, compared to 1:1.79 in those with IgE <200 IU/mL, indicating a higher proportion of males in the group with elevated IgE levels (P=0.017).

The mean age was significantly younger in patients with IgE ≥200 IU/mL than those with IgE <200 IU/mL (29.11 years vs. 33.86 years, P=0.005). However, no significant differences were observed between the two groups regarding AA subtypes, disease severity, disease duration, or involvement of other regions. These results were summarized in **Table 3**.

**Table 3:** Comparison of clinical features on AA patients divided by 200 IU/mL.

Variables	IgE≧200 IU/mL	IgE<200 IU/mL	Test of significance	P value
Age, years ± SD	29.11 ± 14.91	33.86 ± 14.14	U test	0.005*
Gender, n pts (%)				
Male	37(51)	130(36)	Chi-square test	0.017*
Female	36 (49)	233(64)		
Mean AA duration, months ± SD	45.83 ± 68.34	35.83 ± 57.03	U test	0.188
Severity, n pts (%)				
Mild	27(37)	160(44)	Chi-square test	0.264
Moderate to severe	46(63)	203(56)		
AA subtypes, n pts (%)				
LAA	53(73)	288(79)	Chi-square test	0.102
AU/AT	14(19)	34(9)		
ADTA	2 (2)	13(4)		
Ophiasis	4 (4)	28(8)		
Eyebrow/eyelash and nail affection, n pts (%)				
Positive	54 (74)	280(77)	Chi-square test	0.560
Negative	19 (26)	83(23)		

AA, alopecia areata; AU/AT, alopecia universalis or alopecia totalis; ADTA, acute diffuse and total alopecia; LAA, localized alopecia areata.

## 4 Discussion

Clinical characteristics in AA patients demonstrated significant association with disease severity and therapeutic response, including early disease onset and disease durations (12). The severity of AA is not only based on clinical presentation but may also be influenced by the presence of allergic diseases and/or elevated total serum IgE levels, which could indicate a more severe disease (5). IgE demonstrates immunomodulatory capacities through its interaction with mast cells and basophils. IgE promotes the secretion of Th2 cytokines by binding to FcϵRI receptors on the mast cells. In AA patients, mast cell infiltration is observed around hair follicles, and an increase in mast cells and FcϵRI+ cell infiltration was observed in individuals with a history of atopic dermatitis or elevated serum IgE levels (13, 14). The binding mediates IL-4-triggered Treg cell dysfunction and activates localized inflammatory cascades via cytokine networks (15). Treg cells mediate the proliferation and differentiation of hair follicle stem cells, Th2 immune response may reduce the number of Treg cells (16). These findings suggest that IgE may serve as an indirect immunomodulatory role in pathogenesis of AA.。

Elevated activation of IgE-mediated immune pathways has been clinically observed in autoimmune diseases including rheumatoid arthritis, systemic lupus erythematosus and atopic diseases including atopic dermatitis, and asthma (17); however, the correlation between IgE and pathogenesis of AA remain unpredictable. Several studies have observed that the incidence of elevated total serum IgE levels in AA patients ranges from 19.7% to 54% (18–22). In our study, we found that 135 (30.5%) AA patients had elevated total serum IgE levels, similar to the results of O’Loughlin and Kasumagić-Halilović (19, 20). Our study included a larger sample size, with a male-to-female ratio of 1:1.6, which may explain the slightly lower proportion of patients with elevated IgE levels compared to Attia and Bakry’s research (21, 22). We observed that male AA patients had higher IgE levels than female patients. Detailed demographic information is provided in **Table 4**.

**Table 4:** Included researches evaluating total serum IgE levels in AA patients.

Researches	Numbers of patients	Male/Female ratio	Age range(years)	Rate of elevated IgE	Rate in control group
Przybilla^15^	NK	NK	NK	19.7%	NK
O’Loughlin^16^	50	1:0.85	5-58	30%	NK
Kasumagić-Halilović^17^	60	1:0.82	16-77	37%	16%
Attia^18^	54	1:0.50	20-65	48.3%	NK
Bakry^19^	50	1:0.79	10-53	54%	NK
Our research	381	1:1.6	2-74	30.5%	21.0%

NK, Unknown.

A previous study suggested a positive correlation between the severity of AA and Th2 cells in skin tissue, which could explain the relationship between serum IgE levels and disease severity (23). Bakry et al. found that patients with mild AA (n = 17) had higher IgE levels than those with moderate-to-severe AA (n = 28) or diffuse AA (n = 5), which is not significant (22). Our study observed a significant difference in total serum IgE levels between moderate-to-severe AA patients (n = 249) and mild AA patients (n = 187) (129.00 ± 195.90 IU/mL vs. 82.07 ± 99.50 IU/mL, *P*=0.040). The discrepancy between studies may be due to differences in patient numbers.

Regarding disease duration, we observed that patients with a disease duration longer than 2 years had higher serum IgE levels (126.08 ± 221.43 IU/mL) compared to those with a shorter duration (99.76 ± 121.19 IU/mL), however, the difference was not statistically significant. One study reported that peripheral blood CD4+ and CD8+ T cell levels in AA patients were lower than healthy controls, and the CD4^+^/CD8^+^ T cell ratio was positively correlated with disease duration (24). One reason for this outcome is that as the disease progresses, the number of Treg cells decreases at the site of alopecia, leading to an increase in CD4+ and CD8+ T cells around the hair follicles (25, 26). Another reason is that CD4+ and CD8+ immune cells migrate to the affected areas by the expression of chemokine receptors (CKR) on CD4+ and CD8+ T cells (27). The elevated levels of CD4+ T cells can promote the conversion to IgE isotypes, which in turn leads to an increase in IgE levels (28). This may explain the trend of increasing total serum IgE levels with disease progression.

In terms of AA subtypes, we found that patients with AU/AT had significantly higher serum IgE levels compared to those with patchy alopecia (180.79 ± 223.87 IU/mL vs. 98.85 ± 132.15 IU/mL). However, no significant difference was observed in patients with ophiasis and ADTA. One study reported an increased CD4+/CD8+ ratio in AU patients, but not in those with patchy alopecia, but they didn’t observed significantly decrease of CD4 T cells and CD8 T cells in blood (29). Another study reported a patient with AU had normal number of CD4+ T cells but impaired IL-4 production assessed by RT-PCR, and his IgE levels was extremely low (<1 IU/mL) (30). The underlying pathogenesis of total serum IgE level differences among AA subtypes warrants further investigation.

Dupilumab, an IL-4 and IL-13 inhibitor, has demonstrated greater efficacy in AA patients whose IgE levels exceed 200 IU/mL (11). Our study suggests that male patients and children having higher total serum IgE levels than females and adults. Additionally, patients with IgE levels ≥200 IU/mL tended to be younger and had a higher male-to-female ratio than those with <200 IU/mL. One case series reported that 16 of 18 children treated with dupilumab showed hair regrowth, while only 2 worsened (31). However, a separate study observing female patients showed better efficacy than male patients in AA with a history of atopic dermatitis, the difference in dupilumab response can be explained by gender-related differences (31). Dupilumab has demonstrated significant therapeutic efficacy in atopic diseases such as atopic dermatitis (32), but in AA patients few guidelines includes dupilumab as insufficient evidence (33). Based on the findings of our study, clinical stratification of potential responders may be implemented before treatment. In our study, we excluded patients with a history of atopic diseases and did not track treatment outcomes for these patients, the immunological state of non-atopic AA is different from that of atopic AA (34). Therefore, further research is needed to assess the effects of dupilumab in AA patients with or without atopic diseases. Omalizumab is an IgE inhibitor, there was a comparison of effectiveness among omalizumab, mepolizumab and dupilumab in asthma patients (35), however, further clinical trials quantifying therapeutic response of omalizumab on AA patients with high IgE levels are warranted.

Due to the limitations of the retrospective study design, we were unable to analyze the relationship between total serum IgE levels and treatment outcomes or relapses in AA patients. Quantification of allergen-specific IgE could not be universally performed, consequently, total IgE levels were selected as the surrogate biomarker for analysis. Further large-sample-size, prospective studies are urgently needed.

## 5 Conclusion

We observed elevated total serum IgE levels in almost one third of 436 AA patients without atopic diseases. Furthermore, our findings indicate that male patients, children, and those with moderate-to-severe alopecia were more likely to have elevated IgE levels. These results suggest that IgE could be a marker for disease susceptibility and severity in AA. Studies on possible future therapies targeting IgE and type 2 inflammation (such as dupilumab and other Th2 pathway blockers) for the treatment of AA are warranted, especially for AA patients with elevated IgE.

## Data Availability

The original contributions presented in the study are included in the article/supplementary material. Further inquiries can be directed to the corresponding authors.
